# Unraveling the complexity of the associations between students’ science achievement, motivation, and teachers’ feedback

**DOI:** 10.3389/fpsyg.2023.1124189

**Published:** 2023-03-30

**Authors:** Ioannis Katsantonis, Ros McLellan, Pablo E. Torres

**Affiliations:** Faculty of Education, University of Cambridge, Cambridge, United Kingdom

**Keywords:** achievement motivation, motivational beliefs, science achievement, PISA, teachers’ feedback

## Abstract

In recent decades, national science achievement in Greece is following a declining trend. A commonly held assumption is that achievement declines may occur either due to low quality teaching practices or due to students’ low motivation. While motivational beliefs have been linked with achievement, there is not enough evidence connecting these motivational constructs with teachers’ feedback, which can play an important role in nurturing both students’ motivation and achievement. Given that less is known about how these variables collectively function in predicting students’ science achievement, the present study draws upon the Greek (*N* = 5,532 students, *N* = 211 schools) PISA 2015 dataset to address this issue. A serial multiple mediation multilevel structural equation model was deployed. The results illustrated that the association between feedback and science achievement was partially mediated by the complex network of associations between students’ motivational beliefs. Intrinsic motivation was the strongest predictor of achievement, while feedback positively predicted students’ motivational beliefs. Unexpectedly, feedback was a negative predictor of achievement both at the individual and school level. The results suggest that interventions are needed to target specifically teachers’ feedback practices and intrinsic motivation.

## 1. Introduction

A comparative inspection of the time-series of the Greek national achievement in secondary schools indicates a decreasing trend in science achievement (OECD, 2014, 2016a, 2019). While there are many possible factors contributing to this trend (e.g., school, family, peers), it is argued that the most proximal factors influencing achievement, that are of educational and psychological importance, are those related to processes taking place in schools, classrooms and students themselves, such as teaching strategies as well as students’ motivation to learn. More distal factors, such as the structure of the educational system or the national curriculum, are typically stable and beyond the influence of students and teachers in centralized governing systems, such as the Greek one (Kougias and Efstathopoulos, 2020). The psychological processes, though, that are under the explicit control of the learners are malleable to change (Winne and Nesbit, 2010), and, thus, susceptible to psychoeducational interventions. One of the most critical psychological factors affecting students’ learning and achievement is motivational beliefs, such as self-efficacy, intrinsic and extrinsic motivation, and goal orientations (*cf.*, Skaalvik and Skaalvik, 2006; Hulleman et al., 2010; Richardson et al., 2012; Pitsia et al., 2017; Karakolidis et al., 2019; Katsantonis, 2020). Hence, the question remains whether academic achievement can be explained in part by students’ motivational beliefs.

The teaching quality, narrowly defined as strategies/practices implemented by teachers, could also be an explanatory factor of the declines in academic achievement (Schleicher, 2016; Yi and Lee, 2017). In fact, research on teaching effectiveness has posited that teachers’ behaviors and what occurs in classrooms are the most significant factors for explaining student outcomes and the development of metacognitive skills (Caro et al., 2016; Cordero and Gil-Izquierdo, 2018). Among the many teaching strategies documented in the literature, feedback practices seem to be powerfully related to students’ academic motivation (Hattie and Timperley, 2007; Jansen et al., 2022) and achievement (Hattie and Timperley, 2007; Wisniewski et al., 2020). Nevertheless, teachers’ feedback may not always have a positive influence on students’ achievement (Swaffield, 2008) due to various reasons that will be described below. Additionally, empirical evidence is inconclusive regarding the nature of the potential impact of this powerful teaching strategy on students’ motivational beliefs.

Hence, the current study draws upon three main theoretical perspectives, namely the self-determination theory of intrinsic vs. extrinsic motivation (Ryan and Deci, 2000; Ryan and Deci, 2016; Ryan and Deci, 2020), the social-cognitive theory of self-efficacy (Bandura, 1997), and the goal orientation theory (Nicholls, 1984; Ames and Archer, 1988; Ames, 1992). Motivational beliefs, such as self-efficacy, intrinsic and extrinsic motivation, and goal orientations, have been found to be critical factors affecting students’ learning and achievement. Similarly, feedback practices have been identified as a powerful teaching strategy that could potentially improve students’ academic motivation and achievement. However, the nature of the potential impact of feedback on students’ motivational beliefs is still unclear.

In short, the aim is to investigate the relationship between students’ motivational beliefs, teachers’ feedback practices, and science achievement in Greek secondary schools. Specifically, we seek to answer the following overarching research question: How do students’ motivational beliefs and teachers’ feedback practices collectively function as a system to predict science achievement in Greek secondary schools? By examining the relationship between students’ motivational beliefs and teachers’ feedback practices, this study aims to contribute to our understanding of the most effective pathways toward improved science achievement in Greek secondary education.

## 2. Theoretical framework

### 2.1. Complex relationships between students’ motivational beliefs

Although achievement motivational beliefs are many (Eccles and Wigfield, 2002; Wigfield et al., 2021), we attempt to model the relationships between self-efficacy, performance-approach goal orientation, and intrinsic and extrinsic motivation in this study. These motivational beliefs have been postulated as essential components that drive students’ forethought stage of self-regulated learning, with the latter construct encompassing use of cognitive and metacognitive strategies to monitor, control, and regulate learning (Zimmerman and Moylan, 2009; Zimmerman et al., 2017). Understanding how these motivational factors, which were available in the dataset, are linked with feedback practices could have implications for improving students’ self-regulated learning, too. Hence, in this section, we briefly review some of the extant empirical evidence of the links between these motivational beliefs.

Recent empirical evidence illustrated that self-efficacy, defined as personal persuasive judgement of one’s capability to complete an academic task or activity with success (Ferla et al., 2009), was a significant predictor of higher intrinsic motivation (i.e., enjoyment and interest) and lower extrinsic motivation above and beyond demographic influences (McGeown et al., 2014). Similarly, a study with middle school students found that self-efficacy strongly predicted students’ intrinsic motivation which, in turn, predicted effort, persistence, and help-seeking behavior (Skaalvik et al., 2015). Thus, we hypothesize that self-efficacy would predict greater intrinsic (H1) and lower extrinsic motivation (H2).

More complicated appear to be the structural relationships between self-efficacy and goal orientations, defined as the aims/purposes why students engage in learning tasks (Ames and Archer, 1988; Ames, 1992; Elliot et al., 2017; Wigfield et al., 2021). These relationships are mostly undertheorized and underexplored in the empirical literature. The general consensus, though, is that noteworthy correlations exist between self-efficacy and goal orientations (Skaalvik, 1997; Midgley et al., 1998; Ilishkina et al., 2022). What is not unanimously agreed is the directional nature of this relationship. That is, some studies found evidence in favor of a direct pathway from goal orientations to self-efficacy (Midgley et al., 1995; Roeser et al., 1996; Coutinho and Neuman, 2008), while other studies indicated a reverse pathway from self-efficacy to goal orientations as will be discussed below.

According to the trichotomous model of goal orientations (mastery, performance-approach, performance-avoidance; Elliot and Harackiewicz, 1996), perceptions of competence and ability are assumed to be antecedents of mastery (engaging with a task to improve competence) and performance goal orientations (engaging with task to demonstrate competence), where high perceptions of competence predict greater approach goals (mastery and performance-approach), while low perceptions predict greater avoidance goals (performance avoidance; Elliot and Hulleman, 2017). Hence, we follow previous theoretical evidence (Elliot and Hulleman, 2017) and argue that some form of self-awareness is needed before students can opt for a specific goal orientation. Therefore, it could be argued that students’ self-efficacy, as a perception of capabilities, is needed prior to deciding on whether to approach (performance-approach) or avoid a task (performance-avoidance). This theoretical perspective has informed more recent studies. For instance, empirical evidence (Skaalvik and Skaalvik, 2006; Diseth, 2011; Putarek and Pavlin-Bernardić, 2020) illustrated that self-efficacy positively predicted performance-approach goals and mastery goals. Thus, we hypothesize that self-efficacy would predict greater performance-approach goal orientation (H3). Given that empirical research has shown that all the above motivational constructs influence students’ achievement outcomes (Carpenter, 2007; Cellar et al., 2011; Taylor et al., 2014), we expect positive effects on science achievement (H4).

This brief overview lays the groundwork for the current study. Although these motivational psychological variables are firmly grounded in substantial empirical and theoretical evidence, we argue that it is not well established how they function collectively in predicting students’ science achievement and what the nature of the relationships between them may be. Moreover, few empirical studies have examined what the role of teachers’ feedback, as a powerful predictor of achievement itself (Hattie and Timperley, 2007), may be in shaping students’ motivations. Hence, in the following section, we discuss the role of teachers’ feedback in shaping students’ motivation and achievement.

### 2.2. Powerful but controversial effects of feedback

Feedback is usually defined as information given from an agent (i.e., a teacher, in this case) regarding different aspects of one’s (i.e., a student’s) performance (Wisniewski et al., 2020). As will be shown, theoretical accounts of feedback suggest that it influences students’ motivational beliefs and academic achievement. The literature records many types of feedback (e.g., summative, formative, negative, positive, self-referenced, etc.) that influence outcomes differently (Kluger and DeNisi, 1996).

The literature on teaches’ feedback suggests that there a several types of feedback that can be offered to students. For instance, if the feedback is provided for summative assessment (e.g., end of term exam), then it has a judgmental nature, whereas, if the feedback is offered within the framework of formative assessment, then it has a more descriptive nature (Swaffield, 2008). The impact of feedback is not always positive, even though in education it is considered a “good thing” (Swaffield, 2008). In fact, a large-scale meta-analysis of 131 studies revealed that about 40% of the effect sizes documenting the association between feedback and attainment were negative (Kluger and DeNisi, 1996). On the other hand, students themselves may not interpret or incorporate feedback appropriately. An influential review notes that students may not perceive feedback as something positive since may highlight their low competence and/or lack of skills, or they may fail to understand that feedback can act as a helpful guideline (Black and Wiliam, 1998). With respect to the frequency of teachers’ feedback practices in class, it is noted that it is generally low (Hattie and Timperley, 2007). Nevertheless, given that most of the extant evidence indicates a positive association between feedback and achievement, we expect a positive predictive relationship (H5).

With regards to the links between feedback and students’ motivation, the literature suggests that the nature of the relationships between feedback and motivational variables is more complex than it seems. Depending on the target (i.e., self or task) and the nature (i.e., positive or negative) of feedback, Hattie and Clarke (2018) note that it can have a beneficial or detrimental effect on students’ self-efficacy. In general terms, studies have shown that feedback was associated with higher levels of self-efficacy (Chan and Lam, 2010; Duijnhouwer et al., 2010; Abbas and North, 2018). Hence, we hypothesize that feedback would positively predict self-efficacy (H6).

A well-known meta-analysis (Deci et al., 1999) underscored that more positive feedback was positively associated with interest-a component of intrinsic motivation (Ryan and Deci, 2020). A meta-analysis of 78 studies found that negative feedback reduced intrinsic motivation (Fong et al., 2019). A recent experimental study showed that receiving feedback (operationalized as knowledge of results) during a computer-administered task was associated with higher intrinsic motivation (Abbas and North, 2018). Significantly less is known about the relationship between feedback and extrinsic/instrumental motivation. Evidence coming from one experimental study (Oker et al., 2020) and one correlational study (Guo and Wei, 2019) indicated a positive association between feedback and extrinsic motivation. Therefore, it is hypothesized that feedback would predict greater intrinsic and extrinsic motivation (H7).

Finally, the association between feedback with goal orientations is more nuanced given the multifaceted nature of goal orientations. Specifically, a study indicated that self-referential compared to normative feedback positively predicted mastery goals and negatively predicted performance (approach and avoidance) goals (Pekrun et al., 2014). Another study reported that normative feedback (i.e., comparison with others) was linked with more performance-approach goals (Shin et al., 2017). Similarly, an experimental study illustrated that students who received normative feedback endorsed more performance goals (Butler, 2006). Subsequently, it is reasonable to expect that feedback will predict greater performance-approach goals (H8).

In sum, most of the above studies have not established what would be the simultaneous influence of teachers’ feedback on science self-efficacy, performance-approach goal orientation, and intrinsic and extrinsic motivation. Additionally, given that Greek adolescent students are consistently underperforming compared to adolescents from other countries, it raises the question whether the impact of teachers’ feedback is not that positive after all or whether the problem lies with students’ low academic motivation. Hence, the need for further research using robust nationally representative data.

In the following section, we provide a brief overview of the pedagogical and structural characteristics of the Greek context under study.

### 2.3. The Greek educational system at a glance

The Greek educational system is centrally structured. This means that the Ministry of Education and its scientific advisory body called the Institute of Educational Policy (formerly, Pedagogical Institute; Law 3966/2011, 2011) are responsible for the specification of the leadership decisions (e.g., employing teachers, educational funding), the design and implementation of the national curriculum, and the assessment of teaching and supporting staff in schools (Saiti, 2012; Alexopoulos, 2019; Kougias and Efstathopoulos, 2020). In this system, (head-) teachers have limited autonomy to intervene in the decision-making at the school unit (Kougias and Efstathopoulos, 2020).

Regarding the pedagogical content that students are taught, all public and private schools are obliged to follow and implement the national curriculum that has been established in 2003 (Law 21072b/C2, 2003). Students are taught using the same textbooks, which are provided for free.^1^ According to a comparative classification of the educational systems, the Greek educational system follows the same educational model as the French, Spanish, and Italian systems (Bulle, 2011). The curricular organization is following an academic trend which focuses on progression and structured development of instruction of academic subjects, emphasizes theoretical learning through hypothetical-deductive skills and explicit psychological processes (i.e., formal exercises for learning; Bulle, 2011). A trend comparative analysis of the educational systems has also revealed that the Greek system is among the worst performing in academic competence, excellence, inclusion, and social equity (Dominguez-Gil et al., 2022). Consequently, more research is needed in this context to understand how performance may be improved.

### 2.4. The present study

In the current study, we opted for a teaching quality approach. Teaching quality models indicate the importance of teachers’ instructional practices for students’ motivational and attainment outcomes (Fauth et al., 2019, 2020). Therefore, it is of utmost importance to explore the relationships between teachers’ feedback and students’ motivational beliefs and achievement. To this end and informed by the reviewed studies, we identified several evidence gaps in the extant literature. Most of the existing studies have examined the relationships between self-efficacy, intrinsic and extrinsic motivation, performance-approach goal orientation, and teachers’ feedback in isolation, neglecting how all these variables can be connected in a functional system to promote science achievement. Additionally, existing models are to some extent misspecified since they do not holistically include all these beliefs but students may hold multiple motivational beliefs (Pintrich, 2000; Pekrun et al., 2009; Wigfield et al., 2015, 2021). Hence, it is of utmost importance to place all these factors in an integrated framework.

Specifically, the study aims to answer the following research questions:

*RQ1*: How are self-efficacy, performance-approach goals, intrinsic and extrinsic/instrumental motivational beliefs related?

*RQ2*: How are teachers’ feedback, students’ motivational beliefs, and science achievement associated?

**Figure 1:**
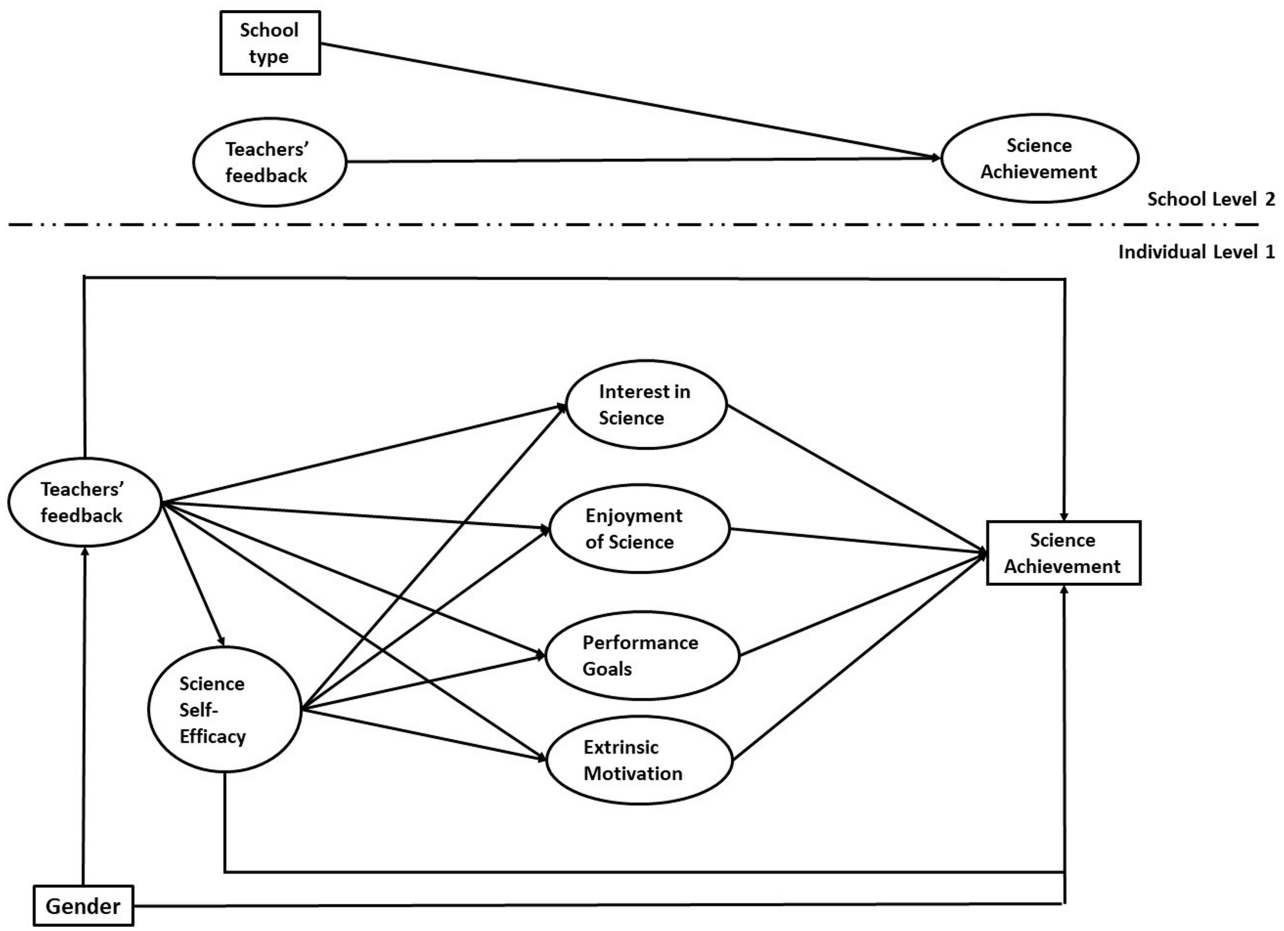
Diagram of the conceptual multilevel SEM model.

As shown in Figure 1, we built and tested a conceptual model that explores the complex structure of the associations between teachers’ feedback, self-efficacy, performance-approach goals, and intrinsic and extrinsic/instrumental motivational beliefs while accounting for school effects. In this model, self-efficacy is hypothesized to predict all other motivational beliefs. Feedback also predicts all motivational beliefs. Motivational beliefs are hypothesized to mediate the association between feedback and science achievement, adjusting for covariates. Overall, it is hoped that the findings could inform teacher training programs or educational policies aimed at improving student achievement and motivation.

## 3. Materials and methods

### 3.1. Dataset and participants

Participants of this study are adolescent students aged 15 years old studying in secondary schools in Greece who participated in the *Programme for International Student Assessment* in 2015 (PISA; OECD, 2016a). This dataset includes a range of motivational beliefs that are not available in previous or later years of the PISA program. The PISA datasets are publicly available for secondary analyses. The Greek PISA sample with complete data on the key measures of interest amounts to 5,532 adolescent students nested in 211 schools. The sample is about equally distributed to gender groups with 48% being males and 52% being females. 95.8% of the students attended public schools and only 4.2% attended private schools.

### 3.2. Measures

All measures were administered in the context of the PISA 2015 testing and were validated through the use of the generalized partial credit Item Response Theory model (IRT) which freely estimates both the difficulty and the discrimination parameters (Muraki, 1992). The scales below show good cross-cultural validity and internal consistency (OECD, 2017). Below, we describe the content and the psychometric properties of the measures. The exact item wordings are available in Supplementary material.

#### 3.2.1. Science self-efficacy

A scale comprising 8 items is indexing students’ efficacy beliefs about their capability in executing science-related tasks (OECD, 2016a). Possible response categories are ranging from 1 “I could do this easily” to 4 “I could not do this.” A sample item is “identify the science question associated with the disposal of garbage.” Item responses were reverse-scored so that higher scores indicate greater levels of SE. Cronbach’s alpha reliability coefficient was 0.86.

#### 3.2.2. Intrinsic motivation-enjoyment and interest in science

Intrinsic motivation is operationalized in this study to comprise enjoyment of science and interest in science. Students were asked how interested they are in several science-related topics. A 5-items measure indexes interest in science and it is scored using a Likert-type scale ranging from 1 “not interested” to 4 “very interested” (OECD, 2016a). The scale’s Cronbach’s reliability coefficient was 0.79. A sample item is “Motion and forces (e.g., Velocity, friction, magnetic and gravitational forces).” The enjoyment of science measure consists of 5 items scored using a Likert-type scale ranging from 1 “strongly disagree” to 4 “strongly agree” (OECD, 2016a). Students were asked how much they (dis) agreed with several statements about their enjoyment of science. A sample item is “I like reading about broad science.” The scale’s Cronbach’s reliability coefficient was 0.93.

#### 3.2.3. Performance-approach goal orientation

PISA 2015 administered a 5-items measure called the “achievement motivation” scale (OECD, 2016b). Students were asked to rate their (dis) agreement with statements such as “I want to be one of the best students in my class.” We argue that this instrument measures performance-approach goal orientation since its items focus on performance, and the self, and describe competence to outperform others. This interpretation is in line with early works on goal orientations (Nicholls, 1984; Ames, 1992). This scale maps onto the performance-approach dimension of the trichotomous model (Elliot and Hulleman, 2017) of goal orientations. Items were scored using a 4-point Likert-type scale ranging from 1 “strongly disagree” to 4 “strongly agree.” The Cronbach’s alpha reliability coefficient was 0.73.

#### 3.2.4. Extrinsic motivation-instrumental motivation

A 4-items scale measures extrinsic motivation (OECD, 2016a). A sample item is “studying my school science subject (s) is worthwhile for me because what I learn will improve my career prospects.” The instrument is scored using a 4-point Likert-type scale ranging from 1 “strongly agree” to 4 “strongly disagree.” The Cronbach’s alpha reliability coefficient was 0.89.

#### 3.2.5. Teachers’ feedback practices

A 5-items scale was administered to tap into students’ perceptions of how frequently they received feedback regarding their learning goals and performance from their science teachers (OECD, 2016b). Feedback was measured in terms of frequency of personal improvement and was more related to task mastery rather than improvement in terms of normative performance. A sample item is “the teacher tells me how I am performing in this course.” The possible item response options are 4 and range from 1 “never or almost never” to 4 “every lesson or almost every lesson.” The Cronbach’s alpha reliability coefficient was 0.90.

#### 3.2.6. Science achievement

PISA 2015 used a standardized balanced incomplete design, where students responded to different but overlapping batteries of science tasks. Afterwards, given the common items, the different subsets of the test were equated using IRT modeling to place students’ scores on the same continuum (OECD, 2017). This procedure is called test equating or linking (Baker, 2001). There is no theoretical minimum or maximum for the PISA achievement score; however, it has been standardized with a mean of 500 and a SD of 100 (OECD, 2019). Given the uncertainty in the computation of students’ science ability estimates, 10 factor scores (called plausible values) were computed for each student, which should be pooled in order to reach valid conclusions (OECD, 2017). The reliability coefficient for the PISA test in Greece was 0.91.

#### 3.2.7. Control variables

Gender was used as a control variable for science achievement and teachers’ feedback. Gender differences in achievement (Yu et al., 2020) and feedback perceptions (Hattie and Timperley, 2007; Henderlong Corpus and Lepper, 2007; Cunha et al., 2019) have been reported in the literature. At school-level, school type (private, government funded private, and public) served as a control given that some literature suggests that public schools may have lower student achievement (Peterson and Llaudet, 2006; Boerema, 2009).

### 3.3. Statistical analyses

To begin with our analyses, confirmatory factor analyses with the WLSMV estimator were performed at the individual level to ascertain the extent to which the scales were displaying internal structure validity (Brown, 2015). Afterwards, the intra-class correlation coefficients were computed to determine whether the variables can be aggregated to the higher level (Heck and Thomas, 2020).

The multilevel serial multiple mediation model was estimated through structural equations under the general structural equation modeling framework in M*plus* 8.7 (Muthén and Muthén, 2017). A structural equation multilevel model with so many ordered-categorical indicators would have been computationally inefficient to estimate due to requiring high dimensional numerical integration (Asparouhov and Muthen, 2007). Hence, summed composite scores were calculated for the motivational variables and feedback, and single-indicator latent factors were formed adjusted for measurement error (1-a reliability; Kline, 2016) so that the multilevel model could be estimated through robust maximum likelihood (MLR). Predictors were grand mean centered in line with methodological guidelines (Hox et al., 2010). Given that 10 plausible values were generated by PISA 2015 per student, we followed existing methodological guidelines (Laukaityte and Wiberg, 2017; Khorramdel et al., 2020) and pooled estimates across all plausible values using Rubin’s rules (Rubin, 2004) in M*plus*.

A bottom-up model-building approach was adopted. In the first step, we estimated a baseline **Model A** with only the level-1 specification (see Figure 1) plus a random intercept for science achievement. Next, the level-2 specification was added with a random intercept for feedback predicting achievement (**Model B**). An alternative **Model C** was estimated to test possible between-school differences in the motivational beliefs. Finally, we tested whether the hypothesized pathway from self-efficacy to performance-approach goals could be reversed (**Model D**).

To evaluate the models’ fit, we used a combination of fit indices. CFI and TLI values close to/above 0.95 in conjunction with RMSEA and SRMR values less than 0.06 are indicating a good model-data fit (Hu and Bentler, 1999). These global fit indices are also applicable to the multilevel SEM, however, they may conceal level-specific misspecification since they describe the degree of fit for the whole model (Ryu and West, 2009). It is noted that in multilevel SEM (MLSEM), the only fit index that is available separately for all levels of the analyses is the SRMR (Silva et al., 2019). Thus, the SRMR values are of particular importance in the multilevel SEM analyses. Moreover, the Bayesian information criterion was also considered, which is more effective in selecting the ‘true’ population model (Bollen et al., 2014). Lower values in the information criteria indicate better model fit and a more parsimonious model (Silva et al., 2019). Given the stratified cluster sampling design implemented by PISA (OECD, 2017), the available sampling weights at both levels, the clustering, and the stratification design information were included in the modeling to adjust the standard errors (TYPE = COMPLEX). The possibility of common method bias was also examined through the Explained Common Variance (ECV) coefficient (Sijtsma, 2009; Rodriguez et al., 2016), which indexes variance explained by a common general latent factor divided by the total variance explained by the group and the general factors (Reise, 2012). ECV values less than 0.80 indicate a multidimensional structure (Rodriguez et al., 2016). The ECV was calculated using the *psych* package (Revelle, 2022) in *R* (R Core Team, 2022).

## 4. Results

### 4.1. Preliminary analyses

In the first instance, the data were subjected to CFAs to determine the extent to which the scales are unidimensional. Modification indices were inspected to identify sources of possible misspecification for improvement of model fit (Kline, 2016). The goodness-of-fit indices of CFA per scale are presented in Table 1.

**Table 1:** Results of construct validity testing.

Scale	Scaled χ^2^ (df)	CFI	TLI	RMSEA	SRMR
SCIEF	424.568^***^ (18)	0.981	0.97	0.065	0.024
PERF	37.706^***^ (4)	0.996	0.991	0.039	0.014
ENJ	44.854^***^ (4)	1	0.999	0.044	0.003
INT	9.358^*^ (3)	1	0.999	0.02	0.005
EXT	14.182^**^ (1)	1	0.997	0.05	0.003
TFEED	63.053^***^ (3)	0.998	0.995	0.062	0.006

^***^p < 0.001; ^**^p < 0.001; ^*^p < 0.05; SCIEF, Science self-efficacy; PERF, Performance-approach goals; ENJ, Enjoyment of science; INT, Interest in science; EXT, Extrinsic motivation; TFEED, Teachers’ feedback.

The values in goodness-of-fit indices in Table 1 indicate that all the scales displayed excellent internal structure validity even with some minor modifications (correlated residuals) due to meaning overlap (Bandalos, 2021). The ECV was equal to 0.36 indicating negligible common method variance (Sijtsma, 2009). Descriptive statistics (Table 2) and bivariate correlations (Table 3) were computed to inspect the distributions and the relationships in the data.

**Table 2:** Descriptive statistics for key variables.

Variable	Mean (SD)	Min-Max	ICC	Total *N*
SCIEF	21.55 (5.5)	8–32	0.025	4,855
PERF	15.05 (2.68)	5–20	0.033	5,320
ENJ	13.50 (4.02)	5–20	0.055	5,144
INT	13.23 (3.75)	5–20	0.082	4,531
EXT	11.51 (3.09)	4–16	0.023	5,129
TFEED	10.54 (4.04)	5–20	0.084	5,023
ACHIEV^a^	454.83 (91.92)	-	0.414	5,532

Weighted descriptive statistics adjusting for non-response and complex sampling design. ^a^All 10 plausible values were pooled; ICC, Intra-class correlation coefficient for level 2; SCIEF, Science self-efficacy; PERF, Performance-approach goals; ENJ, Enjoyment of science; INT, Interest in science; EXT, Extrinsic motivation; TFEED, Teachers’ feedback; ACHIEV, Science achievement.

**Table 3:** Model estimated correlation matrix.

Variable	1	2	3	4	5	6	7
1. SCIEF	1						
2. PERF	0.195	1					
3. ENJ	0.304	0.216	1				
4. INT	0.312	0.203	0.618	1			
5. EXT	0.299	0.181	0.490	0.352	1		
6. TFEED	0.074	*.027^ns^*	0.133	0.125	0.131	1	
7. ACHIEV	0.239	0.189	0.344	0.364	0.138	−0.161	1

All correlations were statistically significant at least at *p* < 0.01; ns, not significant; SCIEF, Science self-efficacy; PERF, Performance-approach goals; ENJ, Enjoyment of science; INT, Interest in science; EXT, Extrinsic motivation; TFEED, Teachers’ feedback; ACHIEV, Science achievement.

As can be seen from the intraclass correlation coefficients (Table 3), science achievement varies significantly across schools (41.4%). Additionally, only the frequency of teachers’ feedback (8.4%) and interest in science (8.2%) and enjoyment of science (5.5%) varied significantly across schools. From the values of the correlation matrix, it can be seen that motivational beliefs are modestly positively correlated among themselves. Most notably, teachers’ feedback and students’ science achievement were negatively correlated.

### 4.2. Unraveling the complexity between science achievement, motivation, and teachers’ feedback

To address the research questions, a multilevel serial multiple mediation model was built and estimated using a bottom-up approach. At the first step, we specified the regression paths at level 1 (as shown in Figure 1) and permitted only a random intercept for science achievement at the school level capturing between-school differences in achievement. This model (**Model A**) had a rather poor fit to the data and especially at the school level, scaled χ^2^(6) = 77.227, *p* < 0.001, CFI = 0.972, TLI = 0.865, RMSEA = 0.046, SRMR_WITHIN_ = 0.016, SRMR_BETWEEN_ = 0.437. In addition, the BIC reached the following values, BIC = 169184.915. In a second model (Model B), a random intercept for teachers’ feedback was added at the school level along with the control variable, and the regression paths to achievement. **Model B** had very good fit at all levels, scaled χ^2^(6) = 53.350, *p* < 0.001, CFI = 0.984, TLI = 0.916, RMSEA = 0.038, SRMR_WITHIN_ = 0.016, SRMR_BETWEEN_ = 0.023. In addition, the BIC confirmed that Model B is improved, BIC = 169072.193. Further covariates (e.g., socio-economic status; immigrant status, etc.) were included at both levels; however, the fit indices rejected those models indicating that these extra variables do not fit well with the theoretical model.

Given that more than 5% of the variance for enjoyment and interest could also be explained by school-level factors (Table 2), a model (C) was estimated with enjoyment and interest as school-level random intercepts predicting between-school differences in achievement. **Model C**’s fit to the data significantly deteriorated, scaled χ^2^(11) = 175.774, *p* < 0.001, CFI = 0.945, TLI = 0.808, RMSEA = 0.052, SRMR_WITHIN_ = 0.01, SRMR_BETWEEN_ = 0.337. Further, the information criteria confirmed this degradation in model-data fit, BIC = 169236.541. Hence, the conceptual model was the best among the alternative models examined.

**Figure 2:**
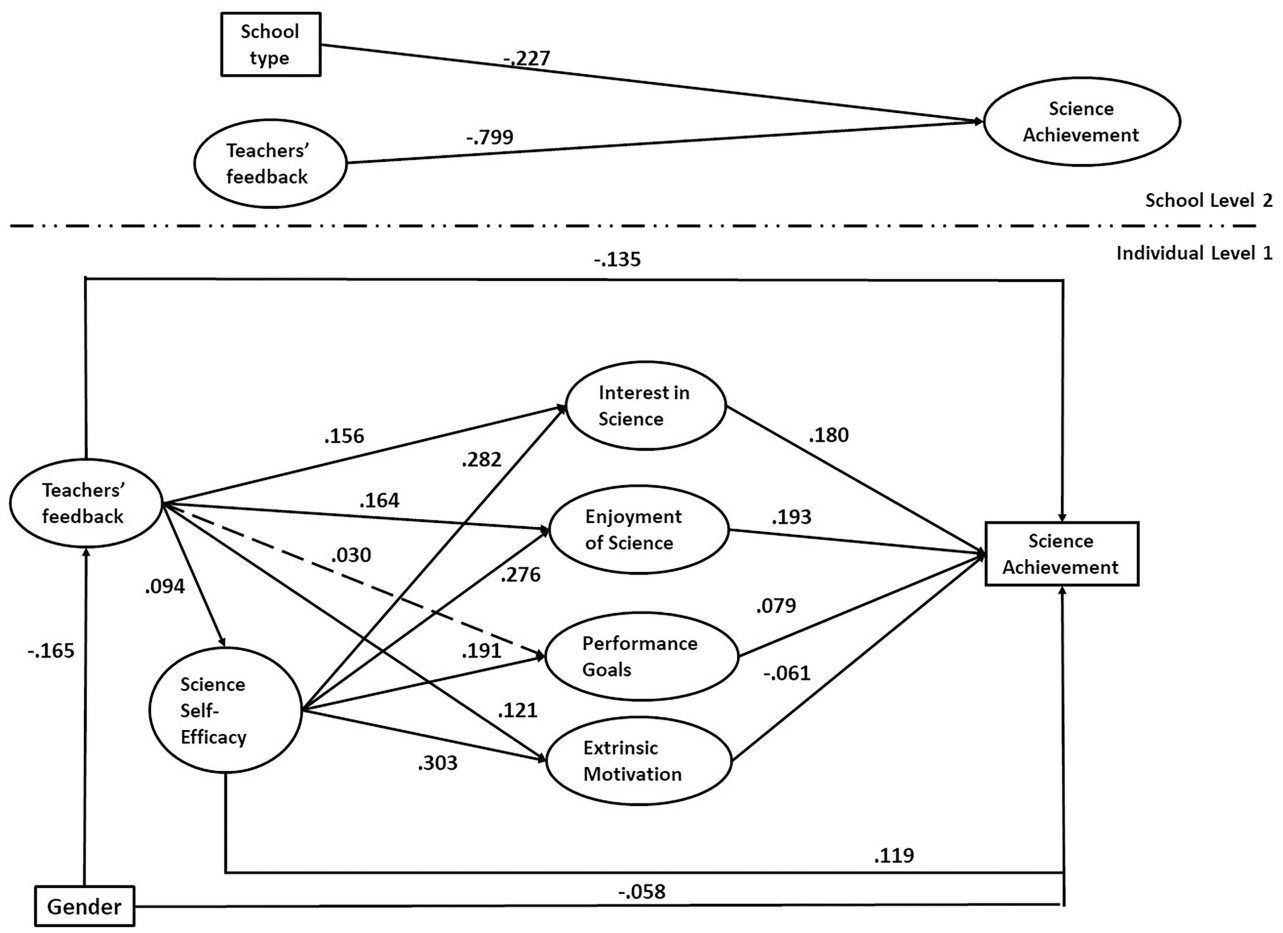
Path diagram of the serial multiple mediation multilevel SEM model (Model B).

In addition to the conceptual model, a nested model (**Model D**) was estimated where performance-approach goal predicted self-efficacy, instead of the reverse. This was done to ascertain the flow of effects given the debate regarding the nature of this relationship. The model with the reverse path was significantly worse fitting to the data variance–covariance matrix and the information criteria confirmed this, scaled χ^2^(9) = 145.828, *p* < 0.001, CFI = 0.953, TLI = 0.837, RMSEA = 0.052, SRMR_WITHIN_ = 0.039, SRMR_BETWEEN_ = 0.023, BIC = 169434.251. Thus, self-efficacy was better represented as predicting performance-approach goals and not the reverse. The models’ fit indices are presented comprehensively in Table 4. Standardized parameter estimates for the conceptual Model B, which displayed the best fit, are shown in Figure 2. Statistically non-significant parameters are depicted with dashed lines. All non-dashed lines represent regression paths that reached statistical significance at least at the 5% level.

**Table 4:** Fit indices for two-level models A–D.

Model	CFI	SRMR within	SRMR between	BIC
Model A	0.972	0.016	0.437	169184.915
**Model B**	**0.984**	**0.016**	**0.023**	**169072.193**
Model C	0.945	0.01	0.337	169236.541
Model D	0.953	0.039	0.023	169434.251

CFI, Comparative Fit Index; SRMR, Standardized Root Mean Residual; BIC, Bayesian Information Criterion. Model A, Level 1 mediation and random-intercept only model for science achievement; Model B (conceptual model), Model A plus the level 2 random intercept of science achievement predicted by feedback and the control; Model C, Model B plus random intercept for enjoyment and interest predicting achievement at level 2; Model D, Model B but reverse pathway from performance-approach goals to self-efficacy. Bold values indicate the best fitting model.

As can be seen in Figure 2, self-efficacy positively predicted science achievement, β = 0.119, *p* < 0.001. Similarly, interest in and enjoyment of science were positive predictors of achievement, β = 0.180, *p* < 0.001; β = 0.193, *p* < 0.001, respectively. Performance-approach goals also were a positive predictor, β = 0.079, *p* < 0.001, whereas, adjusting for the rest of the motivational constructs, extrinsic motivation undermined achievement, β = −0.061, *p < 0*.01. Self-efficacy was a strong positive source for all other motivational constructs with the regression path coefficients ranging from β = 0.191 (performance-approach goals) to β = 0.303 (extrinsic motivation). Higher frequency of teachers’ feedback had beneficial influence on self-efficacy, β = 0.094, *p* < 0.001, enjoyment, β = 0.276, *p* < 0.001, interest, β = 0.156, *p* < 0.001, and extrinsic motivation, β = 0.121, *p* < 0.001, while it had no impact on performance-approach goals, β = 0.030, *p* > 0.05. Surprisingly, higher frequency of teachers’ feedback was associated with lower science achievement both at the student level, β = −0.135, *p* < 0.001, and the school level, β = −0.799, *p* < 0.001. Given that the indirect effects (product terms) were very small, they are not reported.

With respect to the control variables, gender (female) was a negative predictor of both achievement, β = −0.058, *p* < 0.01, and feedback, β = −0.165, *p* < 0.001. Attending public schools was associated with lower achievement between schools compared to private schools, β = −0.227, *p* < 0.001. At the school level, the model explained 69% of the variance of between-school differences in achievement, whereas the model explained 15.3% of the variance of between-student differences at the student level.

## 5. Discussion

Motivated by the declining science performance in Greek secondary schools, the present study, following a pragmatist perspective, sought to clarify the extent to which motivational factors and teachers’ feedback practices may be linked with achievement. Specifically, this study was informed in part by three prominent achievement motivation theories, namely SDT (Ryan and Deci, 2020), social-cognitive theory (Bandura, 1997), and goal orientations theory (Nicholls, 1984; Ames and Archer, 1988). In brief, we aimed to disentangle the complexity between self-efficacy, intrinsic and extrinsic motivation, performance-approach goals, and teachers’ feedback. Hence, the contribution of this study is twofold. Firstly, the theoretical contribution of this work is that we merged parallel strands of achievement motivation research with feedback under a unified framework in predicting science achievement. Secondly, the educational contribution of this study is that through this unified approach it is possible to gain a better understanding of how we should structure successful interventions to increase science achievement.

### 5.1. Associations between motivational beliefs

In greater detail, several hypotheses guided the present research. The first research objective was to estimate the effect of self-efficacy on the other motivational constructs. The MLSEM results illustrated that science self-efficacy (i.e., an academic self-efficacy) was a substantial predictor of greater intrinsic (enjoyment, interest) and extrinsic motivation, and higher performance-approach goals. The positive effect of self-efficacy on intrinsic motivation is a finding which coincides with previous empirical evidence suggesting such associations (McGeown et al., 2014; Skaalvik et al., 2015). Hence, H1 was confirmed. Despite that, higher science self-efficacy was predicting higher extrinsic motivation, which is a unique finding since past evidence suggested that this association was negative (McGeown et al., 2014; Ilishkina et al., 2022). Thus, H2 was rejected. This finding probably depends on the pedagogical context of Greece. Since the context promotes performance structures (value of good normative performance ahead of national exams), then feeling able to do something might mean being motivated to demonstrate good performance. As the model shows this does not necessarily translate to better achievement though, quite the opposite.

Another research hypothesis pertained to the directional nature of the relationship between science self-efficacy and performance-approach goals. What is not well-established is that self-efficacy can predict goal orientations. Most of the preceding empirical studies have indicated that goal orientations predict self-efficacy (Midgley et al., 1995; Roeser et al., 1996; Coutinho and Neuman, 2008), despite the trichotomous model of goal orientations suggesting that perceptions of ability and competence are antecedents of performance-approach goals (Elliot and Harackiewicz, 1996; Elliot and Thrash, 2001; Elliot and Hulleman, 2017). To the best of our knowledge, only three studies have estimated the reverse path (Skaalvik and Skaalvik, 2006; Diseth, 2011; Putarek and Pavlin-Bernardić, 2020). The argument that we put forward here is that a degree of metacognitive judgment of capabilities in science (i.e., science self-efficacy) is needed prior to the adoption of a specific goal orientation. This argument has found empirical support since the MLSEM model D was rejected. This signifies that higher science self-efficacy was predicting greater levels of performance-approach goals (Model B). Hence, H3 was confirmed.

Another objective was to confirm that all motivational constructs would predict students’ science achievement. The modeling (Model B) results underscored the fact that students’ academic motivations were good predictors of achievement. Much can be said about the positive effect of motivation on students’ achievement, but suffice to say that the literature supports the positive links between self-efficacy and academic achievement (Carpenter, 2007; Schunk et al., 2014; Taylor et al., 2014). Although early literature on performance goals has associated performance goals with maladaptive patterns of learning such as surface learning (Anderman and Young, 1994; Midgley et al., 1995; Kaplan and Maehr, 2007), the present results disagree to the extent that performance-approach goals weakly predicted higher science achievement. This result is in agreement with the findings of a large meta-analysis that suggested that performance-approach goals with normative reference were positively, but weakly, associated with academic outcomes (Hulleman et al., 2010). Another finding is that extrinsic motivation was negatively predicting science achievement. Despite that extrinsic and intrinsic motivation are not necessarily antagonistic (Cerasoli et al., 2014) and that a little extrinsic motivation may be needed to be academically flourishing (Lin et al., 2003), the current findings concur with recent empirical literature that found support for the negative consequences of extrinsic motivation on achievement after adjusting for intrinsic motivation (Areepattamannil et al., 2011; Lemos and Veríssimo, 2014; Karlen et al., 2019). Furthermore, the present negative effect of extrinsic/instrumental motivation indicates that the influence of extrinsic motivation on achievement is, indeed, non-additive, adjusting for the rest of the motivational constructs. Overall, H4 was partially supported.

### 5.2. Teachers’ feedback effects on motivational beliefs and science achievement

Another research objective pertained to the role of feedback in promoting science achievement. The present approach differs from preceding evidence since it sought to explore from a multilevel perspective the impact of feedback both between students and between schools. Although extant literature suggests that feedback can usually be a powerful positive predictor of achievement at the individual-level (Hattie and Timperley, 2007; Hattie and Clarke, 2018), the current study found that higher frequency of feedback was predicting lower achievement both between schools and students. This finding is not entirely unprecedented, though, since a large-scale meta-analysis found that about 40% of the associations between feedback and achievement were negative (Kluger and DeNisi, 1996). Nevertheless, the present data do not allow us to identify the source of this controversy. However, this result may point toward deficits in teachers’ feedback strategies (i.e., low quality of feedback) or that feedback is offered exclusively to low-achievers and is not provided to all students. An alternative hypothesis pertaining to the negative nature of this effect is that feedback was provided in an unclear way which induced low performance (Hattie and Timperley, 2007). Additionally, students themselves may not have interpreted feedback positively and, thus, any improvements in achievement may not have been consolidated given that feedback may have been interpreted as judgmental of their (cap-)abilities. This interpretation seems logical given that Black and Wiliam (1998) mention that students may fail to comprehend feedback appropriately. Thus, H5 was rejected.

Additionally, the multilevel modeling showed that feedback was a significant predictor of self-efficacy, intrinsic and extrinsic motivation, but not of performance-approach goals. Thus, H6 and H7 were supported, but H8 was rejected. This finding is in line with preceding evidence indicating that feedback predicted self-efficacy (Chan and Lam, 2010; Duijnhouwer et al., 2010; Hattie and Clarke, 2018) and intrinsic (Deci et al., 1999) and extrinsic/instrumental motivation (Guo and Wei, 2019; Oker et al., 2020). Although previous studies reported that feedback predicted performance-approach goal orientation (Pekrun et al., 2014; Shin et al., 2017), the MLSEM results showed that the path from teachers’ feedback to performance-approach goals did not reach statistical significance. This finding may be linked with the nature of the feedback. Specifically, feedback was measured in terms of frequency of personal improvement, and, thus, was more related to task mastery rather than improvement in terms of normative performance. Hence, this may explain why feedback had no impact on performance-approach goals.

### 5.3. Strengths and limitations

Although the present study is not without any limitations, it should be noted first that it has many strengths. For instance, a large nationally representative sample of about 5,500 students was used, which exceeds the median samples in psychology or education (Kline, 2016). Additionally, the sample was representative of secondary schools’ student population in Greece, which suggests that these findings are generalizable to the wider population. Moreover, the present dataset allowed us to collectively examine the relations between some of the most prominent academic motivational constructs, which is not usually feasible through primary data collection. Further, the multilevel perspective permitted the examination of the true nature of feedback effects at both student and school level. Nevertheless, the present approach was constrained since there were no available data on mastery goals and avoidance performance goals, or the quality and nature of teachers’ feedback practices. Furthermore, the cross-sectional nature of the data did not allow for causal conclusions. More research is also needed with representative sample to ascertain whether the nature of the relationship between feedback and achievement is indeed negative between students and schools. Moreover, the dataset is a little bit outdated, however, since the competence indicators (Dominguez-Gil et al., 2022), the curriculum, and pedagogical structure of the Greek educational system remain relatively stable over time (Kougias and Efstathopoulos, 2020) we could assume that the findings are still pertinent but need to be replicated using other more established measures for cross-validation.

## 6. Conclusion

In conclusion, the above findings indicate that teachers’ feedback and students’ motivation do not work, as well as expected, in a system that brings to bear positive changes in students’ achievement-at least in this context. Although teachers’ feedback had a positive impact on students’ motivational beliefs, this positive impact does not directly translate to increases in students’ science achievement in Greece. This is supported by the inconsistent mediation effects that cancel each other out, resulting in a total effect (MacKinnon et al., 2000) of teachers’ feedback that is zero for achievement. Thus, the feedback’s predictive effects-at least as measured in the current study-are not as powerful as the literature portrayed them to be, instead students’ motivations are more powerful.

### 6.1. Implications for educational policy and practice

Despite any limitations of this work, the findings have potential implications for educational policy and practice. Specifically, the MLSEM revealed that intrinsic motivation and, especially, enjoyment of science, was the most beneficial predictor of science achievement in Greece. Therefore, the national curriculum’s focus could be shifted more toward enhancing students’ enjoyment of science. Additionally, teachers should invest in new methods that would make the content knowledge more enjoyable and interesting to the students. Of course, these suggestions would require a shift from the traditional instructionist model of teaching and the stage-like structured teaching of academic subjects implemented in Greek schools (Bulle, 2011). Our findings may have implications for the Italian, Spanish, and French educational systems that follow similar curricular and pedagogical structure as the Greek educational model (see Bulle, 2011). In order to improve science achievement, we also recommend more evidence-based interventions that place emphasis on both cognitive (e.g., self-efficacy) and emotional (e.g., enjoyment, interest, goal orientation) motivational forces since our results indicated that these had a positive effect on achievement. In contrast, parents and/or teachers should make an effort to restrict explicit instrumental expectations since extrinsic/instrumental motivation has a deleterious effect on achievement.

Beyond students’ motivation, greater emphasis should be placed on promoting highly qualitative teachers’ feedback practices. The present findings suggest that high frequency of feedback was linked with lower achievement, at least in this context. This would suggest the need for further teacher training that would place emphasis on appropriate strategies for the delivery of feedback, especially since secondary school teachers in Greece may not have taken any pedagogical courses (Bista et al., 2016). Feedback was generally found to be low; however, students may not be able to recognize or appreciate feedback. Thus, we recommend explicit provision of constructive feedback targeted specifically at the students’ work. Offered feedback should also not be negative, controlling or uninformative, but should be highly informative and focused both on areas for improvement and specific strategies to improve students’ learning (Wisniewski et al., 2020).

## Data availability statement

The original contributions presented in the study are included in the article/Supplementary material, further inquiries can be directed to the corresponding author.

## Ethics statement

The studies involving human participants were reviewed and approved by Psychology and Education Ethics Committee of the Faculty of Education, University of Cambridge, United Kingdom (29/7/2022). Written informed consent to participate in this study was provided by the participants’ legal guardian/next of kin.

## Author contributions

IK: conceptualization, methodology, formal analysis, resources, data curation, writing—original draft, writing—review and editing, visualization, funding acquisition, and project administration. RM and PT: supervision. All authors contributed to the article and approved the submitted version.

## Funding

IK was supported by a scholarship from the Alexander S. Onassis Foundation (scholarship ID: F ZR 024/1-2021/2022) and has received an educational grant from the A.G. Leventis Foundation.

## Conflict of interest

The authors declare that the research was conducted in the absence of any commercial or financial relationships that could be construed as a potential conflict of interest.

## Publisher’s note

All claims expressed in this article are solely those of the authors and do not necessarily represent those of their affiliated organizations, or those of the publisher, the editors and the reviewers. Any product that may be evaluated in this article, or claim that may be made by its manufacturer, is not guaranteed or endorsed by the publisher.

## Footnotes

^1^ http://ebooks.edu.gr/ebooks/v2/allmaterial.jsp

## References

[ref1] Abbas Z. A. North J. S. (2018). Good-vs. poor-trial feedback in motor learning: the role of self-efficacy and intrinsic motivation across levels of task difficulty. Learn. Instr. 55, 105–112. doi: 10.1016/j.learninstruc.2017.09.009

[ref2] Alexopoulos N. (2019). Resolving school staffing problems in Greece: a strategic management approach. Front. Educ. 4, 1–11. doi: 10.3389/feduc.2019.00130

[ref3] Ames C. (1992). Classrooms: goals, structures, and student motivation. J. Educ. Psychol. 84, 261–271. doi: 10.1037/0022-0663.84.3.261

[ref4] Ames C. Archer J. (1988). Achievement goals in the classroom: students’ learning strategies and motivation processes. J. Educ. Psychol. 80, 260–267. doi: 10.1037/0022-0663.80.3.260

[ref5] Anderman E. M. Young A. J. (1994). Motivation and strategy use in science: individual differences and classroom effects. J. Res. Sci. Teach. 31, 811–831. doi: 10.1002/tea.3660310805

[ref6] Areepattamannil S. Freeman J. G. Klinger D. A. (2011). Intrinsic motivation, extrinsic motivation, and academic achievement among Indian adolescents in Canada and India. Soc. Psychol. Educ. 14, 427–439. doi: 10.1007/s11218-011-9155-1

[ref7] Asparouhov T. Muthen B. (2007). Computationally efficient estimation of multilevel high-dimensional latent variable models. In: *Proceedings of the 2007 JSM meeting in Salt Lake City, Utah, Section on Statistics in Epidemiology*, 2531–2535. Available at: https://citeseerx.ist.psu.edu/viewdoc/download?doi=10.1.1.310.3825&rep=rep1&type=pdf

[ref8] Baker F. B. (2001). The basics of item response theory. 2nd *Edn.* College Park, UMD: ERIC.

[ref9] Bandalos D. L. (2021). Item meaning and order as causes of correlated residuals in confirmatory factor analysis. Struct. Equ. Model. Multidiscip. J. 28, 903–913. doi: 10.1080/10705511.2021.1916395

[ref10] Bandura A. (1997). Self-efficacy: The exercise of control. New York, NY: W H Freeman.

[ref11] Bista P. Kokkinos T. Markoglou A. (2016). Απόψεις μελλοντικών εκπαιδευτικών για τη διδακτική κατάρτιση και παιδαγωγική επάρκεια [Opinions of future educators for teaching training and pedagogical competence]. Sci. Educ. 2, 117–137. doi: 10.26248/.v2016i2.1208

[ref12] Black P. Wiliam D. (1998). Assessment and classroom learning. Assess. Educ. 5, 7–74. doi: 10.1080/0969595980050102

[ref13] Boerema A. J. (2009). Does Mission matter? An analysis of private school achievement differences. J. School Choice 3, 112–137. doi: 10.1080/15582150902996708

[ref14] Bollen K. A. Harden J. J. Ray S. Zavisca J. (2014). BIC and alternative Bayesian information criteria in the selection of structural equation models. Struct. Equ. Model. Multidiscip. J. 21, 1–19. doi: 10.1080/10705511.2014.856691, PMID: 31360054 PMC6663110

[ref15] Brown T. A. (2015). Confirmatory factor analysis for applied research (second edition). New York, NY: The Guilford Press.

[ref16] Bulle N. (2011). Comparing OECD educational models through the prism of PISA. Comput. Educ. 47, 503–521. doi: 10.1080/03050068.2011.555117

[ref17] Butler R. (2006). Are mastery and ability goals both adaptive? Evaluation, initial goal construction and the quality of task engagement. Br. J. Educ. Psychol. 76, 595–611. doi: 10.1348/000709905X52319, PMID: 16953964

[ref18] Caro D. H. Lenkeit J. Kyriakides L. (2016). Teaching strategies and differential effectiveness across learning contexts: evidence from PISA 2012. Stud. Educ. Eval. 49, 30–41. doi: 10.1016/j.stueduc.2016.03.005

[ref19] Carpenter S. L. (2007). A comparison of the relationships of students’ self-efficacy, goal orientation, and achievement across grade levels: A meta-analysis Unpublished Masters Thesis. Simon Fraser University.

[ref20] Cellar D. F. Stuhlmacher A. F. Young S. K. Fisher D. M. Adair C. K. Haynes S.. (2011). Trait goal orientation, self-regulation, and performance: a meta-analysis. J. Bus. Psychol. 26, 467–483. doi: 10.1007/s10869-010-9201-6

[ref21] Cerasoli C. P. Nicklin J. M. Ford M. T. (2014). Intrinsic motivation and extrinsic incentives jointly predict performance: a 40-year meta-analysis. Psychol. Bull. 140, 980–1008. doi: 10.1037/a0035661, PMID: 24491020

[ref22] Chan J. C. Y. Lam S. (2010). Effects of different evaluative feedback on students’ self-efficacy in learning. Instr. Sci. 38, 37–58. doi: 10.1007/s11251-008-9077-2

[ref23] Cordero J. M. Gil-Izquierdo M. (2018). The effect of teaching strategies on student achievement: an analysis using TALIS-PISA-link. J. Policy Model. 40, 1313–1331. doi: 10.1016/j.jpolmod.2018.04.003

[ref24] Coutinho S. A. Neuman G. (2008). A model of metacognition, achievement goal orientation, learning style and self-efficacy. Learn. Environ. Res. 11, 131–151. doi: 10.1007/s10984-008-9042-7

[ref25] Cunha J. Rosário P. Núñez J. C. Vallejo G. Martins J. Högemann J. (2019). Does teacher homework feedback matter to 6th graders’ school engagement?: a mixed methods study. Metacogn. Learn. 14, 89–129. doi: 10.1007/s11409-019-09200-z

[ref26] Deci E. L. Koestner R. Ryan R. M. (1999). A meta-analytic review of experiments examining the effects of extrinsic rewards on intrinsic motivation. Psychol. Bull. 125, 627–668. doi: 10.1037/0033-2909.125.6.627, PMID: 10589297

[ref27] Diseth Å. (2011). Self-efficacy, goal orientations and learning strategies as mediators between preceding and subsequent academic achievement. Learn. Individ. Differ. 21, 191–195. doi: 10.1016/j.lindif.2011.01.003

[ref28] Dominguez-Gil C. Segovia-Gonzalez M. M. Contreras I. (2022). A multiplicative composite indicator to evaluate educational systems in OECD countries. Compare 52, 1296–1313. doi: 10.1080/03057925.2020.1865791

[ref29] Duijnhouwer H. Prins F. J. Stokking K. M. (2010). Progress feedback effects on students’ writing mastery goal, self-efficacy beliefs, and performance. Educ. Res. Eval. 16, 53–74. doi: 10.1080/13803611003711393

[ref30] Eccles J. S. Wigfield A. (2002). Motivational beliefs, values, and goals. Annu. Rev. Psychol. 53, 109–132. doi: 10.1146/annurev.psych.53.100901.135153 11752481

[ref31] Elliot A. J. Dweck C. S. Yeager D. S. (Eds.) (2017). Handbook of competence and motivation: Theory and application. 2nd Edn. New York, NY: Guilford Press.

[ref32] Elliot A. J. Harackiewicz J. M. (1996). Approach and avoidance achievement goals and intrinsic motivation: a mediational analysis. J. Pers. Soc. Psychol. 70, 461–475. doi: 10.1037/0022-3514.70.3.461 8014838

[ref33] Elliot A. J. Hulleman C. S. (2017). “Achievement Goals” in Handbook of competence and motivation: Theory and practice. eds. Elliot A. J. Dweck C. S. Yeager D. S.. 2nd *Edn.* (New York, NY: The Guilford Press).

[ref34] Elliot A. J. Thrash T. M. (2001). Achievement goals and the hierarchical model of achievement motivation. Educ. Psychol. Rev. 13, 139–156. doi: 10.1023/A:1009057102306

[ref35] Fauth B. Decristan J. Decker A.-T. Büttner G. Hardy I. Klieme E.. (2019). The effects of teacher competence on student outcomes in elementary science education: the mediating role of teaching quality. Teach. Teach. Educ. 86:102882. doi: 10.1016/j.tate.2019.102882

[ref36] Fauth B. Wagner W. Bertram C. Göllner R. Roloff J. Lüdtke O.. (2020). Don’t blame the teacher? The need to account for classroom characteristics in evaluations of teaching quality. J. Educ. Psychol. 112, 1284–1302. doi: 10.1037/edu0000416

[ref37] Ferla J. Valcke M. Cai Y. (2009). Academic self-efficacy and academic self-concept: reconsidering structural relationships. Learn. Individ. Differ. 19, 499–505. doi: 10.1016/j.lindif.2009.05.004

[ref38] Fong C. J. Patall E. A. Vasquez A. C. Stautberg S. (2019). A meta-analysis of negative feedback on intrinsic motivation. Educ. Psychol. Rev. 31, 121–162. doi: 10.1007/s10648-018-9446-6

[ref39] Guo W. Wei J. (2019). Teacher feedback and students’ self-regulated learning in mathematics: a study of Chinese secondary students. Asia Pac. Educ. Res. 28, 265–275. doi: 10.1007/s40299-019-00434-8

[ref40] Hattie J. Clarke S. (2018). Visible learning: Feedback. New York, NY: Routledge.

[ref41] Hattie J. Timperley H. (2007). The power of feedback. Rev. Educ. Res. 77, 81–112. doi: 10.3102/003465430298487

[ref42] Heck R. H. Thomas S. L. (2020). “An introduction to multilevel modeling techniques” in MLM and SEM approaches. 4th ed (New York, NY: Routledge).

[ref43] Henderlong Corpus J. Lepper M. R. (2007). The effects of person versus performance praise on Children’s motivation: gender and age as moderating factors. Educ. Psychol. 27, 487–508. doi: 10.1080/01443410601159852

[ref44] Hox J. J. Moerbeek M. van de Schoot R. (2010). Multilevel analysis: Techniques and applications. 2nd *Edn.* New York, NY: Routledge.

[ref45] Hu L. Bentler P. M. (1999). Cutoff criteria for fit indexes in covariance structure analysis: conventional criteria versus new alternatives. Struct. Equ. Modeling 6, 1–55. doi: 10.1080/10705519909540118

[ref46] Hulleman C. S. Schrager S. M. Bodmann S. M. Harackiewicz J. M. (2010). A meta-analytic review of achievement goal measures: different labels for the same constructs or different constructs with similar labels? Psychol. Bull. 136, 422–449. doi: 10.1037/a0018947 20438145

[ref47] Ilishkina D. I. de Bruin A. Podolskiy A. I. Volk M. I. van Merriënboer J. J. G. (2022). Understanding self-regulated learning through the lens of motivation: motivational regulation strategies vary with students’ motives. Int. J. Educ. Res. 113:101956. doi: 10.1016/j.ijer.2022.101956

[ref48] Jansen T. Meyer J. Wig A. (2022). Which student and instructional variables are Most strongly related to academic motivation in K-12 education? A systematic review of meta-analyses. Psychol. Bull. 148, 1–26. doi: 10.1037/bul0000354

[ref49] Kaplan A. Maehr M. L. (2007). The contributions and prospects of goal orientation theory. Educ. Psychol. Rev. 19, 141–184. doi: 10.1007/s10648-006-9012-5

[ref50] Karakolidis A. Pitsia V. Emvalotis A. (2019). The case of high motivation and low achievement in science: what is the role of students’ epistemic beliefs? Int. J. Sci. Educ. 41, 1457–1474. doi: 10.1080/09500693.2019.1612121

[ref51] Karlen Y. Suter F. Hirt C. Maag Merki K. (2019). The role of implicit theories in students’ grit, achievement goals, intrinsic and extrinsic motivation, and achievement in the context of a long-term challenging task. Learn. Individ. Differ. 74:101757. doi: 10.1016/j.lindif.2019.101757

[ref52] Katsantonis I. (2020). Self-regulated learning and reading comprehension: the effects of gender, motivation and metacognition. Hell. J. Psychol. 17, 286–307. doi: 10.26262/hjp.v17i3.7835

[ref53] Khorramdel L. von Davier M. Gonzalez E. Yamamoto K. (2020). “Plausible values: principles of item response theory and multiple imputations” in Large-Scale Cognitive Assessment. eds. D. B. Maehler and B. Rammstedt (Cham: Springer), 27–47.

[ref54] Kline R. B. (2016). Principles and practice of structural equation modeling. 4th *Edn.* New York, NY: Guilford Press.

[ref55] Kluger A. N. DeNisi A. (1996). The effects of feedback interventions on performance: a historical review, a meta-analysis, and a preliminary feedback intervention theory. Psychol. Bull. 119, 254–284. doi: 10.1037/0033-2909.119.2.254

[ref56] Kougias K. Efstathopoulos J. (2020). The operational framework of the Greek educational system as an obstacle to the implementation of sustainable school. Front. Educ. 5, 1–14. doi: 10.3389/feduc.2020.00142

[ref57] Laukaityte I. Wiberg M. (2017). Using plausible values in secondary analysis in large-scale assessments. Commun. Stat. Theory Methods 46, 11341–11357. doi: 10.1080/03610926.2016.1267764

[ref58] Law 21072b/C2. (2003). Διαθεματικό Ενιαίο Πλαίσιο Προγραμμάτων Σπουδών (Δ.Ε.Π.Π.Σ.) και Αναλυτικά Προγράμματα Σπουδών (Α.Π.Σ.) Δημοτικού και Γυμνασίου [Interdisciplinary Common Framework of Programme of Studies (D.E.P.P.S.) and Curriculum (A.P.S.). Greek Government Newsletter. Available at: http://www.pi-schools.gr/download/programs/depps/fek304.pdf

[ref59] Law 3966/2011. (2011). Θεσμικό πλαίσιο των Πρότυπων Πειραματικών Σχολείων, Ίδρυση Ινστιτούτου Εκπαιδευτικής Πολιτικής, Οργάνωση του Ινστιτούτου Τεχνολογίας Υπολογιστών και Εκδόσεων "ΔΙΟΦΑΝΤΟΣ" και Λοιπές διατάξεις [Legal framework of the Experimental Schools, Founding of the Institute of Educational Policy, Organisation of the Institute of Technology, Computers and Printing "Diofantos" and other provisions]. Greek Government Newsletter. Available at: https://www.kodiko.gr/nomothesia/document/125088/nomos-3966-2011

[ref60] Lemos M. S. Veríssimo L. (2014). The relationships between intrinsic motivation, extrinsic motivation, and achievement, along elementary school. Procedia. Soc. Behav. Sci. 112, 930–938. doi: 10.1016/j.sbspro.2014.01.1251

[ref61] Lin Y.-G. McKeachie W. J. Kim Y. C. (2003). College student intrinsic and/or extrinsic motivation and learning. Learn. Individ. Differ. 13, 251–258. doi: 10.1016/S1041-6080(02)00092-4

[ref62] MacKinnon D. P. Krull J. L. Lockwood C. M. (2000). Equivalence of the mediation, confounding and suppression effect. Prev. Sci. 1, 173–181. doi: 10.1023/A:1026595011371, PMID: 11523746 PMC2819361

[ref63] McGeown S. P. Putwain D. Geijer Simpson E. Boffey E. Markham J. Vince A. (2014). Predictors of adolescents’ academic motivation: personality, self-efficacy and adolescents’ characteristics. Learn. Individ. Differ. 32, 278–286. doi: 10.1016/j.lindif.2014.03.022

[ref64] Midgley C. Anderman E. Hicks L. (1995). Differences between elementary and middle school teachers and students: a goal theory approach. J. Early Adolesc. 15, 90–113. doi: 10.1177/0272431695015001006

[ref65] Midgley C. Kaplan A. Middleton M. Maehr M. L. Urdan T. Anderman L. H.. (1998). The development and validation of scales assessing students’ achievement goal orientations. Contemp. Educ. Psychol. 23, 113–131. doi: 10.1006/ceps.1998.0965, PMID: 9576837

[ref66] Muraki E. (1992). A generalized partial credit model: application of an EM algorithm. ETS Res. Rep. Series 1992, 1–30. doi: 10.1002/j.2333-8504.1992.tb01436.x

[ref67] Muthén L. K. Muthén B. O. (2017). Mplus User’s Guide. 8th *Edn.* Los Angeles, CA: Muthén & Muthén.

[ref68] Nicholls J. G. (1984). Achievement motivation: conceptions of ability, subjective experience, task choice, and performance. Psychol. Rev. 91, 328–346. doi: 10.1037/0033-295X.91.3.328

[ref69] OECD. (2014). PISA 2012 results: What students know and can do-student performance in mathematics, Reading and Science. OECD Publishing.

[ref70] OECD (2016a). PISA 2015 results (volume I): excellence and equity in education. Paris: OECD.

[ref71] OECD (2016b). PISA 2015 results (volume II): policies and practices for successful schools. Paris: OECD.

[ref72] OECD. (2017). PISA 2015 technical report. Available at: https://www.oecd.org/pisa/data/2015-technical-report/

[ref73] OECD (2019). PISA 2018 results (volume I): what students know and can do. Paris: OECD.

[ref74] Oker A. Pecune F. Declercq C. (2020). Virtual tutor and pupil interaction: a study of empathic feedback as extrinsic motivation for learning. Educ. Inf. Technol. 25, 3643–3658. doi: 10.1007/s10639-020-10123-5

[ref75] Pekrun R. Cusack A. Murayama K. Elliot A. J. Thomas K. (2014). The power of anticipated feedback: effects on students’ achievement goals and achievement emotions. Learn. Instr. 29, 115–124. doi: 10.1016/j.learninstruc.2013.09.002

[ref76] Pekrun R. Elliot A. J. Maier M. A. (2009). Achievement goals and achievement emotions: testing a model of their joint relations with academic performance. J. Educ. Psychol. 101, 115–135. doi: 10.1037/a0013383

[ref77] Peterson P. E. Llaudet E. (2006). “On the public-private school achievement debate” in Paper prepared for the annual meetings of the American Political Science Association Philadelphia, PA, August 2006. Available at: https://scholar.harvard.edu/files/ellaudet/files/on_the_public-private_school_achievement_debate.pdf

[ref78] Pintrich P. R. (2000). Multiple goals, multiple pathways: the role of goal orientation in learning and achievement. J. Educ. Psychol. 92, 544–555. doi: 10.1037/0022-0663.92.3.544

[ref79] Pitsia V. Biggart A. Karakolidis A. (2017). The role of students’ self-beliefs, motivation and attitudes in predicting mathematics achievement: a multilevel analysis of the Programme for international student assessment data. Learn. Individ. Differ. 55, 163–173. doi: 10.1016/j.lindif.2017.03.014

[ref80] Putarek V. Pavlin-Bernardić N. (2020). The role of self-efficacy for self-regulated learning, achievement goals, and engagement in academic cheating. Eur. J. Psychol. Educ. 35, 647–671. doi: 10.1007/s10212-019-00443-7

[ref81] R Core Team. (2022). R: A language and environment for statistical computing. R Foundation for Statistical Computing. Available at: https://www.R-project.org/

[ref82] Reise S. P. (2012). The rediscovery of Bifactor measurement models. Multivar. Behav. Res. 47, 667–696. doi: 10.1080/00273171.2012.715555, PMID: 24049214 PMC3773879

[ref83] Revelle W. (2022). Psych: Procedures for psychological, psychometric, and personality research. Northwestern University. Available at: https://CRAN.R-project.org/package=psych Version = 2.2.5.

[ref84] Richardson M. Abraham C. Bond R. (2012). Psychological correlates of university students’ academic performance: a systematic review and meta-analysis. Psychol. Bull. 138, 353–387. doi: 10.1037/a0026838, PMID: 22352812

[ref85] Rodriguez A. Reise S. P. Haviland M. G. (2016). Evaluating bifactor models: calculating and interpreting statistical indices. Psychol. Methods 21, 137–150. doi: 10.1037/met0000045, PMID: 26523435

[ref86] Roeser R. W. Midgley C. Urdan T. C. (1996). Perceptions of the school psychological environment and early adolescents’ psychological and behavioral functioning in school: the mediating role of goals and belonging. J. Educ. Psychol. 88, 408–422. doi: 10.1037/0022-0663.88.3.408

[ref87] Rubin D. B. (2004). Multiple imputation for nonresponse in surveys. Hoboken, NJ: Wiley-Interscience.

[ref01] Ryan R. M. Deci E. L. (2000). Intrinsic and extrinsic motivations: Classic definitions and new directions. Contem. Educ. Psychol. 25, 54–67. doi: 10.1006/ceps.1999.1020 10620381

[ref02] Ryan R. M. Deci E. L. (2016). Facilitating and hindering motivation, learning, and well-being in schools. New York, NY: Routledge Handbooks Online. doi: 10.4324/9781315773384.ch6

[ref88] Ryan R. M. Deci E. L. (2020). Intrinsic and extrinsic motivation from a self-determination theory perspective: definitions, theory, practices, and future directions. Contemp. Educ. Psychol. 61:101860. doi: 10.1016/j.cedpsych.2020.101860

[ref89] Ryu E. West S. G. (2009). Level-specific evaluation of model fit in multilevel structural equation modeling. Struct. Equ. Model. Multidiscip. J. 16, 583–601. doi: 10.1080/10705510903203466

[ref90] Saiti A. (2012). Leadership and quality management: an analysis of three key features of the Greek education system. Qual. Assur. Educ. 20, 110–138. doi: 10.1108/09684881211219370

[ref91] Schleicher A. (2016). Teaching excellence through professional learning and policy reform: lessons from around the world. Paris: OECD.

[ref92] Schunk D. H. Meece J. L. Pintrich P. (2014). Motivation in education theory, research, and applications. Upper Saddle River, NJ: Pearson.

[ref93] Shin J. Lee Y. Seo E. (2017). The effects of feedback on students’ achievement goals: interaction between reference of comparison and regulatory focus. Learn. Instr. 49, 21–31. doi: 10.1016/j.learninstruc.2016.11.008

[ref94] Sijtsma K. (2009). On the use, the misuse, and the very limited usefulness of Cronbach’s alpha. Psychometrika 74, 107–120. doi: 10.1007/s11336-008-9101-0, PMID: 20037639 PMC2792363

[ref95] Silva B. C. Bosancianu C. M. Littvay L. (2019). Multilevel structural equation modeling. 1st *Edn.* Thousand Oaks, CA: SAGE Publications, Inc.

[ref96] Skaalvik E. M. (1997). Self-enhancing and self-defeating ego orientation: relations with task and avoidance orientation, achievement, self-perceptions, and anxiety. J. Educ. Psychol. 89, 71–81. doi: 10.1037/0022-0663.89.1.71

[ref97] Skaalvik E. M. Federici R. A. Klassen R. M. (2015). Mathematics achievement and self-efficacy: relations with motivation for mathematics. Int. J. Educ. Res. 72, 129–136. doi: 10.1016/j.ijer.2015.06.008

[ref98] Skaalvik E. M. Skaalvik S. (2006). “Self-concept and self-efficacy in mathematics: relation with mathematics motivation and achievement” in The concept of self in education, family and sports. ed. Prescott A. (New York, NY: Nova Science Publishers), 51–74.

[ref99] Swaffield S. (Ed.) (2008). Unlocking assessment: Understanding for reflection and application. New York, NY: Routledge.

[ref100] Taylor G. Jungert T. Mageau G. A. Schattke K. Dedic H. Rosenfield S.. (2014). A self-determination theory approach to predicting school achievement over time: the unique role of intrinsic motivation. Contemp. Educ. Psychol. 39, 342–358. doi: 10.1016/j.cedpsych.2014.08.002

[ref101] Wigfield A. Eccles J. S. Fredricks J. A. Simpkins S. Roeser R. W. Schiefele U. (2015). “Development of achievement motivation and engagement” in Handbook of child psychology and developmental science. ed. Lerner R. (New York, NY: John Wiley & Sons), 1–44.

[ref102] Wigfield A. Muenks K. Eccles J. S. (2021). Achievement motivation: what we know and where we are going. Ann. Rev. Dev. Psychol. 3, 87–111. doi: 10.1146/annurev-devpsych-050720-103500

[ref103] Winne P. H. Nesbit J. C. (2010). The psychology of academic achievement. Annu. Rev. Psychol. 61, 653–678. doi: 10.1146/annurev.psych.093008.100348 19575616

[ref104] Wisniewski B. Zierer K. Hattie J. (2020). The power of feedback revisited: a meta-analysis of educational feedback research. Front. Psychol. 10, 1–14. doi: 10.3389/fpsyg.2019.03087, PMID: 32038429 PMC6987456

[ref105] Yi H. S. Lee Y. (2017). A latent profile analysis and structural equation modeling of the instructional quality of mathematics classrooms based on the PISA 2012 results of Korea and Singapore. Asia Pac. Educ. Rev. 18, 23–39. doi: 10.1007/s12564-016-9455-4

[ref106] Yu J. McLellan R. Winter L. (2020). Which boys and which girls are falling behind? Linking adolescents’ gender role profiles to motivation, engagement, and achievement. J. Youth Adolesc. 50, 336–352. doi: 10.1007/s10964-020-01293-z, PMID: 32734562 PMC7875942

[ref107] Zimmerman B. J. Moylan A. R. (2009). “Self-regulation: where metacognition and motivation intersect” in Handbook of metacognition in education. eds. D. J. Hacker, J. Dunlosky and A. C. Graesser (New York, NY: Routledge/Taylor & Francis Group), 299–315.

[ref108] Zimmerman B. J. Schunk D. H. DiBenedetto M. K. (2017). “The role of self-efficacy and related beliefs in self-regulation of learning and performance” in Handbook of competence and motivation. eds. Elliot A. J. Dweck C. S. Yeager D. S.. 2nd *Edn.* (New York, NY: Guilford Press), 313–333.

