# Supplementary material for: Unraveling the complexity of the associations between students’ science achievement, motivation, and teachers’ feedback
Source: Front Psychol. 2023 Mar 30;14:1124189. doi: 10.3389/fpsyg.2023.1124189 (PMC10097932; doi:10.3389/fpsyg.2023.1124189)
Supplement: Supplementary file 1 [file Table_1.docx]

Supplementary Material

Unravelling the Complexity of the Associations between Students’ Science Achievement, Motivation, and Teachers’ Feedback

Ioannis Katsantonis*, Ros McLellan, Pablo E. Torres

*** Correspondence:** Ioannis Katsantonis: [ik388@cam.ac.uk](mailto:ik388@cam.ac.uk)

Measures’ Item Wordings

Item Wordings for PISA 2015 Scales (OECD, 2016a)

Science Self-Efficacy Scale

How easy do you think it would be for you to perform the following tasks on your own?

| 1. recognise the science question that underlies a newspaper report on a health issue |
| --- |
| 1. explain why earthquakes occur more frequently in some areas than in others |
| 1. describe the role of antibiotics in the treatment of disease |
| 1. identify the science question associated with the disposal of garbage |
| 1. predict how changes to an environment will affect the survival of certain species |
| 1. interpret the scientific information provided on the labelling of food items |
| 1. discuss how new evidence can lead you to change your understanding about the possibility of life on Mars |
| 1. identify the better of two explanations for the formation of acid rain |

Intrinsic Motivation- Interest in Science

To what extent are you interested in the following <broad science> topics?

| 1. Biosphere (e.g. Ecosystem services, sustainability) |
| --- |
| 1. Motion and forces (e.g. Velocity, friction, magnetic and gravitational forces) |
| 1. Energy and its transformation (e.g. Conservation, chemical reactions) |
| 1. The Universe and its history |
| 1. How science can help us prevent disease |

Intrinsic Motivation- Enjoyment of Science

How much do you disagree or agree with the statements about yourself below?

| 1. I generally have fun when I am learning <broad science> topics |
| --- |
| 1. I like reading about <broad science> |
| 1. I am happy working on <broad science> topics |
| 1. I enjoy acquiring new knowledge in <broad science> |
| 1. I am interested in learning about <broad science> |

Achievement Motivation- Performance-Approach Goal Orientation

To what extent do you disagree or agree with the following statements about yourself?

| 1. I want top grades in most or all of my courses |
| --- |
| 1. I want to be able to select from among the best opportunities available when I graduate |
| 1. I want to be the best, whatever I do |
| 1. I see myself as an ambitious person |
| 1. I want to be one of the best students in my class |

Instrumental Motivation- Extrinsic Motivation

How much do you agree with the statements below?

| 1. Making an effort in my <school science> subject(s) is worth it because this will help me in the work I want to do later on |
| --- |
| 1. What I learn in my <school science> subject(s) is important for me because I need this for what I want to do later on |
| 1. Studying my <school science> subject(s) is worthwhile for me because what I learn will improve my career prospects |
| 1. Many things I learn in my <school science> subject(s) will help me to get a job |

Teachers’ Feedback Practices

How often do these things happen in your lessons for this <school science> course?

| 1. The teacher tells me how I am performing in this course |
| --- |
| 1. The teacher gives me feedback on my strengths in this <school science> subject |
| 1. The teacher tells me in which areas I can still improve |
| 1. The teacher tells me how I can improve my performance |
| 1. The teacher advises me on how to reach my learning goals |
